# Investigation of magnetic droplet solitons using x-ray holography with extended references

**DOI:** 10.1038/s41598-018-29856-y

**Published:** 2018-08-01

**Authors:** E. Burgos-Parra, N. Bukin, S. Sani, A. I. Figueroa, G. Beutier, M. Dupraz, S. Chung, P. Dürrenfeld, Q. Tuan Le, S. M. Mohseni, A. Houshang, S. A. Cavill, R. J. Hicken, J. Åkerman, G. van der Laan, F. Y. Ogrin

**Affiliations:** 1 College of Engineering, Mathematics and Physical sciences, University of Exeter, EX4 4QL Exeter, United Kingdom; 2 Department of Materials and Nanophysics, School of Engineering Sciences, KTH Royal Institute of Technology, 164 40 Kista, Sweden; 3 Magnetic Spectroscopy Group, Diamond Light Source, Didcot, United Kingdom; 4 University Grenoble Alpes, CNRS, Genoble INP, SIMaP, Grenoble, France; 5 Department of Physics, University of Gothenburg, 412 96 Gothenburg, Sweden; 6 Department of Physics and Astronomy, Uppsala University, 751 20 Uppsala, Sweden; 7 Faculty of Physics, Shahid Beheshti University, Evin, 19839 Tehran Iran; 8 NanOsc AB, Electrum 205, 164 40 Kista, Sweden; 9 Department of Physics, University of York, YO10 5DD York, United Kingdom

## Abstract

A dissipative magnetic soliton, or magnetic droplet, is a structure that has been predicted to exist within a thin magnetic layer when non-linearity is balanced by dispersion, and a driving force counteracts the inherent damping of the spin precession. Such a soliton can be formed beneath a nano-contact (NC) that delivers a large spin-polarized current density into a magnetic layer with perpendicular magnetic anisotropy. Although the existence of droplets has been confirmed from electrical measurements and by micromagnetic simulations, only a few attempts have been made to directly observe the magnetic landscape that sustains these structures, and then only for a restricted set of experimental parameter values. In this work we use and x-ray holography technique HERALDO, to image the magnetic structure of the [Co/Ni]x4 multilayer within a NC orthogonal pseudo spin-valve, for different range of magnetic fields and injected electric currents. The magnetic configuration imaged at −33 mA and 0.3 T for devices with 90 nm NC diameter reveals a structure that is within the range of current where the droplet soliton exist based on our electrical measurements and have it is consistent with the expected size of the droplet (∼100 nm diameter) and its spatial position within the sample. We also report the magnetisation configurations observed at lower DC currents in the presence of fields (0–50 mT), where it is expected to observe regimes of the unstable droplet formation.

## Introduction

A typical spin valve structure is a trilayer film consisting of a magnetic pinned layer (PL), a non-magnetic spacer layer, and a magnetic free layer (FL). The FL is magnetically softer than the PL and therefore more able to change its magnetisation state when small magnetics fields are applied. The main attraction of spin valves is that a relative change in the orientation of the magnetisation in the FL and PL produces large changes in the resistance due the giant magnetoresistance effect, making it ideal for sensing very small magnetic fields^1^. However, when large electrical current densities (≥10^7^ A/m^2^) are applied, these devices can also behave as spin transfer oscillators (STOs)^2^. In a STO a DC electrical current may be converted to a microwave signal when the angular momentum carried by a spin polarized current exerts a torque on the magnetisation of the FL. As was first shown by Slonczewski^3,4^ this spin transfer torque (STT) can then lead either to the suppression of the damping in the FL and therefore a persistent precession of the magnetisation vector, or to a total reversal of the magnetisation. In order to obtain the large current density needed, point-contacts are often used to inject the current into the multilayer sample stack. The size of such point contacts ranges from tens to hundreds of nanometres, in what is known as a nano-contact spin transfer oscillator (NC-STO). In a NC-STO the NC is positioned on the top of the FL. In the orthogonal NC-STO^5,6^ studied here, the magnetisations of the PL and FL are almost completely orthogonal to each other in the remanent state. While the PL magnetisation lies in plane, the FL has perpendicular magnetic anisotropy so that its magnetisation points out of the plane. The STT associated with the injected DC current excites sustained and stable oscillations of the magnetisation in an area localized beneath the NC. Due to its high tunability, high quality factor, and the ease by which it can be combined with conventional semiconductor technology, the NC-STO promises to be a corner stone for the development of future technologies such as magnetic memories, microwave nano-emitters, and neuromorphic computing among others^7^.

The NC-STO provides an ideal environment in which to nucleate a dissipative magnetic soliton, which we refer to as a droplet from here on. A droplet is a localized excitation sustained by STT, with magnetisation opposite of the rest of the free layer, for which the amplitude of precession decays exponentially towards its boundaries^8^. Similar to theoretically predicted conservative solitons^9^, magnetic droplets can be sustained by balancing exchange with anisotropy energies and through the opposing effects of the damping and STT.

In 2013, an experimental study led by J. Åkerman^10^ showed that a magnetic droplet can be created and maintained within a NC-STO. Further investigations based on transport measurements and micro-magnetic simulations of these systems^11–20^ revealed a rich dynamic structure, promising a range of applications in microwave technology. However, few experimental techniques allow the magnetisation of these structures to be imaged with adequate resolution (≤32 nm)^21,22^ while the necessary external magnetic field is applied. Therefore only a few direct images have been obtained^23–25^ of magnetic solitons confined with NC-STO devices, so that the different dynamic regimes of the system remain largely unexplored.

In the present work we report the first direct measurement of the magnetic structures in the FL of an orthogonal NC-STO for different combinations of external magnetic field and applied DC current. In order to obtain magnetic information from the sample we combined holography with extended reference autocorrelation by linear differential operator (HERALDO)^26,27^ with the magnetic contrast provided by X-ray magnetic circular dichroism (XMCD). The latter, being element-specific, allows probing different layers separately. Our implementation of magnetic HERALDO allows to access both the perpendicular and parallel components of the magnetization^21,22,28,29^. Here we work in perpendicular X-ray incidence geometry, which provides the perpendicular component of magnetization^28^. A particular advantage in this case is also that the magnetisation can be imaged beneath the electrical contacts. Studies of the magnetisation both around and underneath the nano-contact are crucial for understanding the importance of the effects produced by Oersted field in the nucleation and subsequent drift of the droplets. In this study the sample fabrication process does not allow the exact location of the NC to be known, however as we show below, the area of the NC can be identified by the unique structure of the domains nucleated by the Oersted field of the DC current. Imaging the magnetic structure at different values of the magnetic field and electric current shows a significant variation in the domain structure around the NC. We demonstrate that at certain conditions, correlated with the intensity spectra observed in transport measurements, a localised magnetic structure with opposite polarisation to surrounding magnetisation can be observed. We suggest that this visualisation is in direct agreement with magnetic droplet formation at the field and current values expected from electrical measurements.

## Results

**Figure 1:**
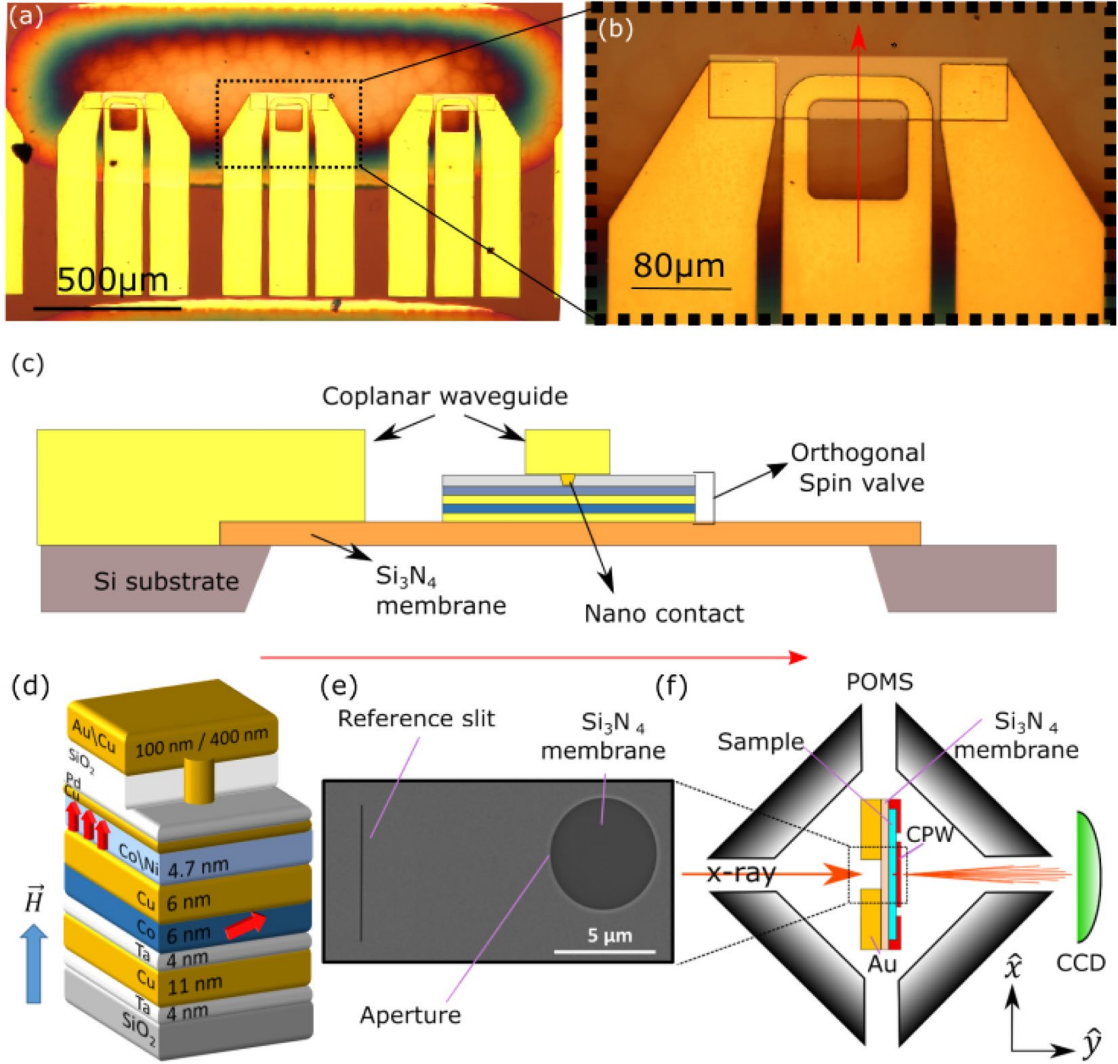
(**a**) Set of three coplanar waveguide used for HERALDO. The oval shaped region is the Si3N4 membrane. (**b**) Zoom of the top section a CPW shown in (a). The red arrow depicts the position where the transversal cut shown in (**c**). (**c**) Schematic of a transversal cut along the red arrow in (**b**) where the position of the nano contact is shown. (**d**) Schematic of a section of the 16 × 8 *μ*m^2^ mesa layer containing the nano-contact orthogonal pseudo spin-valve, where the Co/Ni multilayer acts as the free layer and the Co layer as the pinned layer. In this work devices with Cu nano-contacs of 90 and 110 nm diameter were studied. The red arrows indicate the magnetization of the magnetic layers after appliying a magnetic field ranging within 20–3000 mT out-of-plane (blue arrow). (**e**) Au layer covering one side of the Si_3_N_4_ membrane. An aperture of 5 *μ*m diameter and a reference slit of 6 *μ*m in length and ∼60 nm width were milled using a focused ion beam. The pseudo spin-valve is located on the opposite side of the Si_3_N_4_ membrane (**f**) Schematic set up for HERALDO measurements with an external magnetic field. The sample is positioned in the middle of a portable octupole magnet system (POMS) and the coherent x-rays from the synchrotron source pass through the aperture and the reference slit. The resulting diffraction pattern is captured by a CCD camera at a distance ∼60 cm behind the sample, at the end of the beam-line. The coplanar waveguide (CPW) supplies the DC current that passes through the magnetic layers and generates the STT required to form the droplet soliton.

Electrical transport measurements and time-averaged imaging of the dynamics of the out-of-plane magnetisation within an orthogonal NC-STO were performed for samples with NC diameter of 90 nm and 110 nm for different combinations of applied magnetic field and DC injected current. Figure 1(a) and (b) shows the coplanar waveguide deposited on the top of the multilayer stack and used for transport measurements on these devices. Figure 1(c) and (d) shows an schematic of the multi-layered device on top of the S_3_N_4_ membrane and the intended position of the NC within the sample. The fabrication, electrical characterization, and HERALDO measurements carried out on these samples are described in detail in the Methods section.

### Transport measurements

**Figure 2:**
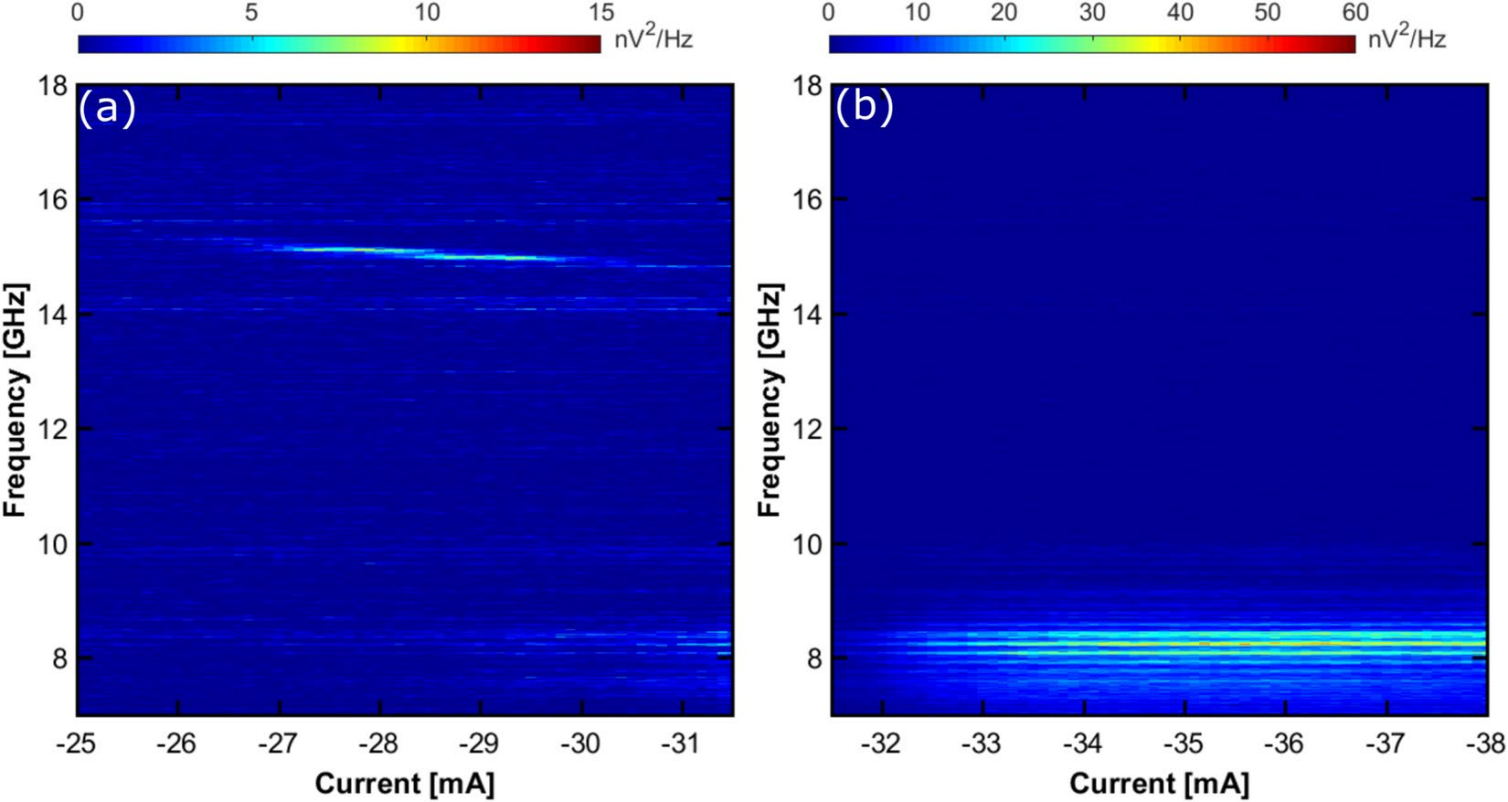
Power spectral density (PSD) is represented by the colour scale for an orthogonal pseudo spin-valve with a nano-contact of 90 nm diameter with a magnetic field of 0.4 T applied perpendicular to the sample plane. When the threshold current for the nucleation of the droplet is reached (∼−30.5 mA), a sudden drop in in the emission frequency of ∼8 GHz occurs in addition to a dramatic increase of the emitted power, as is shown in (**a**) and (**b**). The additional structure is an artifact due to standing waves in one or more of the cables between the microwave components in the measurement chain.

Figure 2 shows the power spectral density (PSD) with a ∼−0.4 T external magnetic field applied to a device with 90 nm nano-contact diameter. Microwave emission occurs at a frequency of 15 ± 0.1 GHz from −27 to −30.5 mA, which then drops to 8.2 GHz, as the emitted power increases by a factor of about 5. Furthermore, emission at a lower frequency of a few hundreds of MHz is also detected once the threshold current for the nucleation of the droplet is reached (not shown here). This is in agreement with previous results where the appearance of lower frequency (hundreds of MHz) dynamics, simultaneous drop in frequency and an increase in emitted power of the principal microwave mode occurred as a droplet was formed^14,30^. Considering also the stability map presented in reference^14^, the device with a 90 nm NC diameter is a good candidate for imaging current-induced magnetisation dynamics in a range of magnetic fields. The device with 110 nm NC diameter also revealed similar electrical features but the magnetic field needed to image a magnetic droplet lies outside of the range that could be achieved within the used experimental set-up, which was 0.35 T.

### Holographic measurements

Figure 1(e) shows a SEM image of a standard sample used in HERALDO measurements. In this case, the device is built on one side of a Si_3_N_4_ membrane while the other side of the membrane is covered by a Au layer of ∼600 nm thickness. A reference slit and an aperture are milled into the Au layer using a focused ion beam, which together generate the diffraction pattern produced by coherent x-rays passing through and captured by the CCD camera, as shown in the schematic of the set-up in Fig. 1(f).

**Figure 3:**
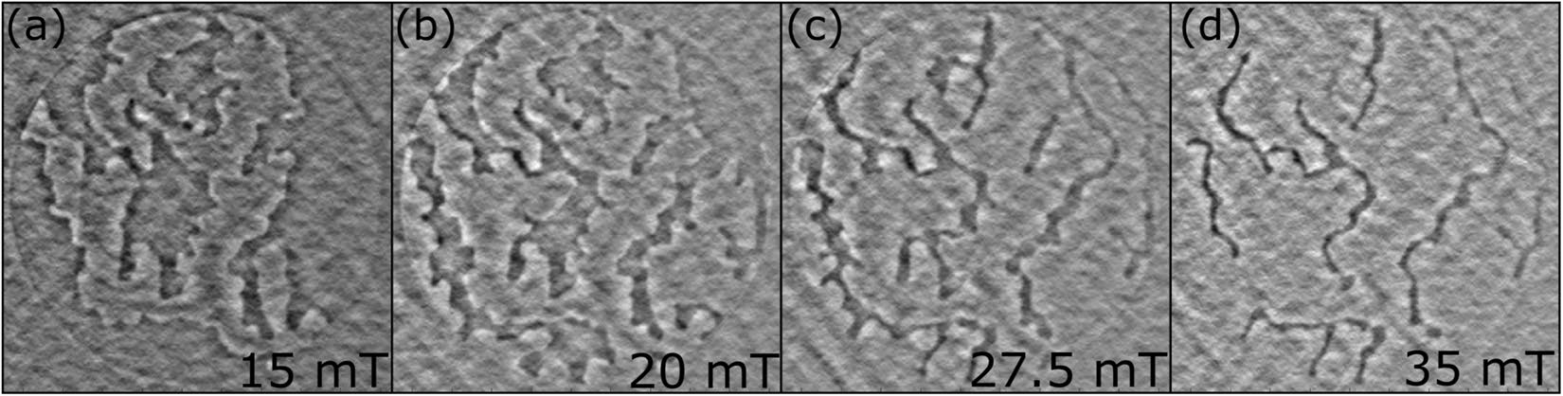
Magnetic domains formed on Ni within the free layer on the orthogonal pseudo spin-valve. The sample was saturated in the $-\hat{y}$ direction before an external magnetic field was applied in the +$\hat{y}$ direction (defined in inset of Fig. 1(c)) with a magnitude of (**a**) 15 mT, (**b**) 20 mT, (**c**) 27.5 mT, and (**d**) 35 mT. The brighter (darker) regions correspond to magnetisation with a component in the $\hat{y}$ ($-\hat{y}$) direction. Bright regions overcome dark regions as expected as the magnetic field saturates the sample magnetisation in the $+\hat{y}$ direction.

The magnetic domain configurations found within both samples at zero current are the characteristic maze-like domain pattern well described in the literature for samples with perpendicular magnetic anisotropy (PMA). Figure 3 shows four magnetic domain patterns corresponding to four different points on the hysteresis curve. It is evident how the the balance between magnetic domains of opposite polarisation changes as the magnetic field is increased. Even though the total thickness of the Ni layer is only ∼3.2 nm, and imaged behind a 500 nm copper contact, the magnetic contrast is remarkably good. It is worth to notice that the magnetic contrast of smaller domains is more pronounced. Larger domains appear more ‘grey-ish’ and non-uniform. This effect is related to some limitation of the set-up, but not undermining the nature of the observations. Generally, due to the use of a beam-stop, which partially blocks some of the scattered light, some information is lost, specifically in the low-angle scattering, which would normally be associated with larger objects. Smaller scatters, such as narrower magnetic domains, would lead to larger angle diffraction not affected by the beam-stop^31^.

**Figure 4:**
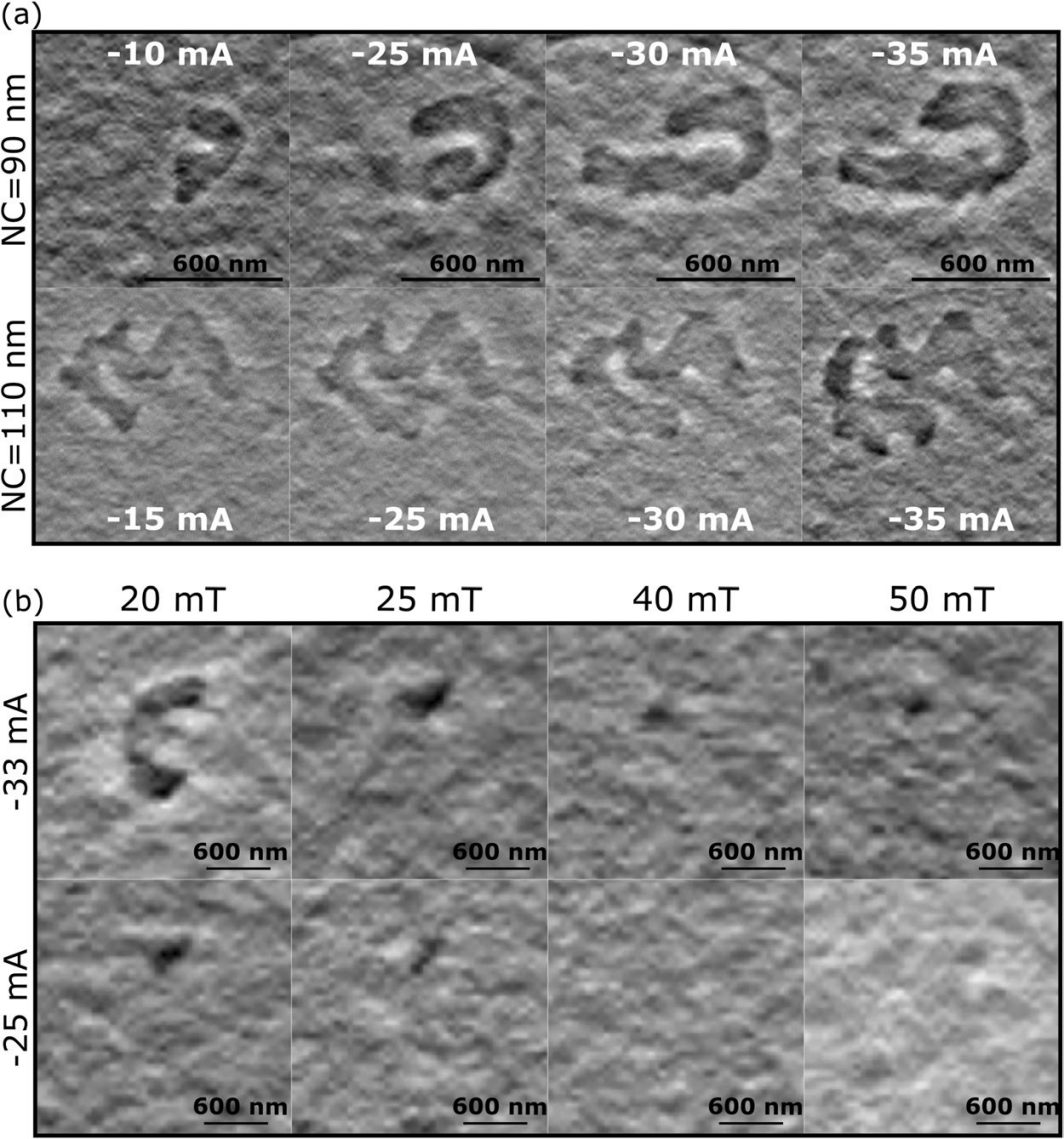
Magnetic structures observed for different fields and applied current values. The samples were saturated by an out-of-plane field of 0.05 T that was then reduced to zero. (**a**) The darker regions are the magnetic domains created by passing a DC current of −10, −15, −25, −30 and −35 mA through NCs of 90 (upper panel) and 110 nm diameter (lower panel) with zero external magnetic field. The nucleation of the domain is followed by small increments in its size as the amplitude of the DC current is increased. (**b**) The samples were magnetically saturated at 0.05 T and the field was then removed. The DC current injected through the 110 nm NC was fixed at values of −33 (upper panel) and −25 mA (lower panel) and HERALDO measurements were made with fields of 20, 25, 40 and 50 mT applied perpendicular to the sample plane in the direction of the initial saturation, parallel to the direction of the x-rays. The domain size decreases as the external magnetic field is increased, vanishing when the external magnetic field is sufficient to overcome the influence of the Oersted field produced by the current.

An out-of-plane external field of 0.05 T was first applied to magnetically saturate the Co/Ni multilayer. DC currents ranging from −10 to −35 mA were then injected into the nano-contact at zero external magnetic field. It was observed that a single magnetic domain formed with magnetisation opposite to that of the rest of the sample. It is not possible to locate the exact position of the NC by optical or electronic means after the device is finished, but the location of the NC, and hence the maximum Oersted field generated by the current, is expected to be within the region where the reverse domains are nucleated. For the sample with 90 nm (110 nm) NC diameter this reverse domain first appears at −10 mA (−15 mA). Figure 4(a) shows how this domain grows in size as the amplitude of the DC current is increased. Due to the size of the magnetic domain formed, we suggest that its growth is driven by the Oersted field generated by the current passing through the NC. This field is large enough to locally modify the magnetisation as shown in Fig. 4. This effect was also seen for positive current values. In contrast, the STT is not expected to contribute to domain formation outside of the NC region.

Afterwards, to magnetically saturate the FL again, an out-of-plane field of 0.05 T was applied and then removed. A constant current of −33 mA was then applied to the sample with a 110 nm NC diameter at zero field. Small fields ranging from 20 to 50 mT were applied in the same out-of-plane direction to explore the interplay between the current and the external applied field. Under these conditions, a small C-shaped domain is observed at 20 mT that we infer to be nucleated from the NC. As the magnetic field was increased, the magnetic domain collapsed to a small region that was observed through all field steps. The same experiment performed instead with a current of −25 mA injected into the 110 nm contact (Fig. 4(b), bottom panel) showed a similar domain to that observed before at 25 mT and −33 mA, but in this case at 20 mT. This reverse domain vanished when the magnetic field was increased to 30 mT, such that the sample appeared to be in a state of uniform magnetic saturation (Fig. 4(b), bottom panel for 40 and 50 mT).

Due to the available size and orientation of the magnetic field, all the former measurements were made on devices with a PL magnetized mostly in plane and a FL magnetically saturated out of plane. It was shown above that, for a NC of 110 nm diameter, the Oersted field generated by a current of −33 mA nucleated a reverse domain at zero field within a saturated sample, as shown in Fig. 4. When an external field is applied, this effect is still visible but the size of the domain nucleated by the current is smaller than for the zero field case. As the external field is increased, it begins to dominate and the effect of the Oersted field is only seen at shorter distances from the nano-contact. The interplay between the external field and the Oersted field produced by the DC current modifies the local magnetic free energy, and therefore the reverse domain might still appear even for fields where the FL would otherwise be saturated, as can be seen in Fig. 4(b).

**Figure 5:**
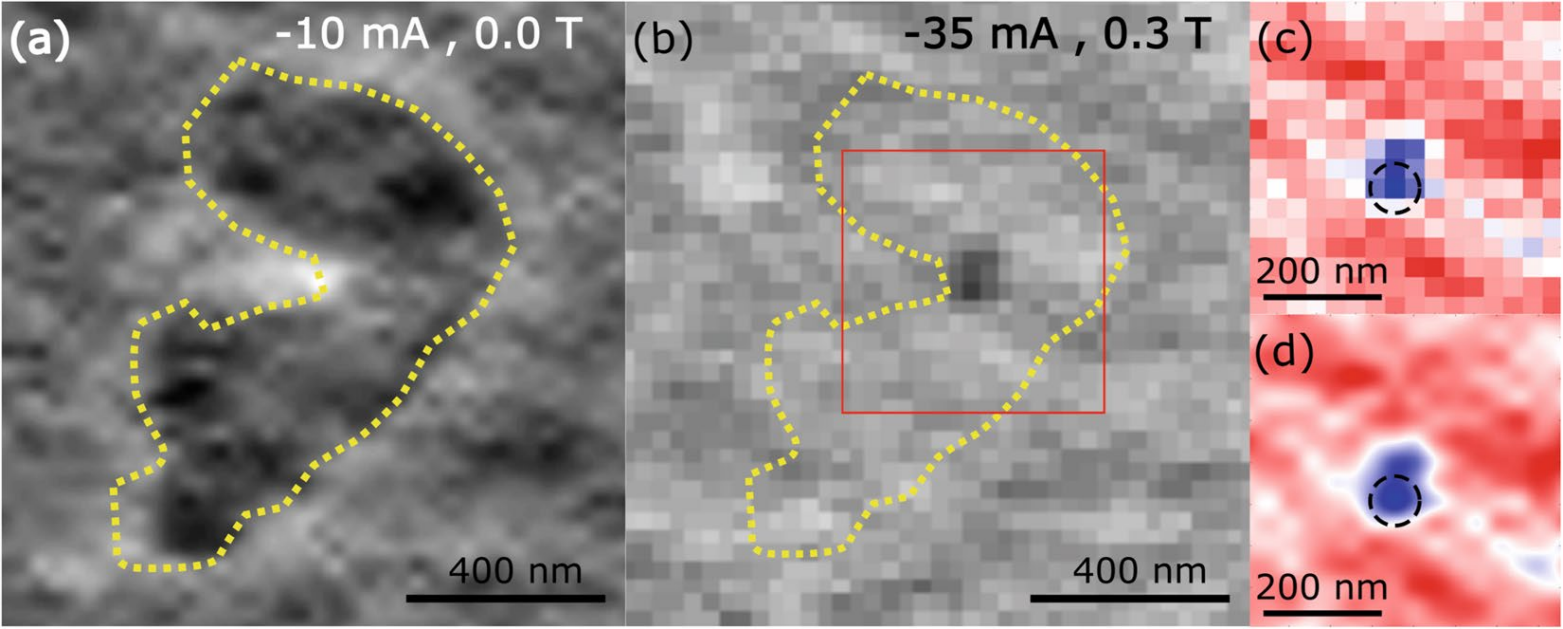
(**a**) Details of the magnetic contrast observed for the 90 nm NC sample for −10 mA injected current at zero external field shown in Fig. 4(a), top left. The dashed line denotes the edge of the domain generated by the DC current. (**b**) The magnetic contrast obtained for −35 mA injected current and 0.3 T applied perpendicular to the sample is shown. The dashed line from (a) has been superimposed for comparison of the size and position of the magnetic features in (a) and (b). The black region in (b) is ascribed to a magnetic droplet soliton nucleated under the NC. The region enclosed by the red square in (b) is shown in (**c**) with a different colour scale, and in (**d**) after an interpolation and smoothing process. The black dashed line in (c) and (d) has 90 nm diameter and shows the suggested position of the NC within the sample. The centre of the NC was taken to be at the pixel with the greatest intensity.

In order to work within the magnetic field range where transport measurements showed the nucleation of a droplet, an external field of −0.3 T was applied perpendicular to the plane of the sample with a 90 nm diameter NC. A localized reversal of the magnetisation was observed at −35 mA injected current at the location inferred for the NC (Fig. 5 (b). This structure was not present in the images taken at smaller current values, while the value of −35 mA is in good agreement with that expected (∼−33 m(a), from theory and transport measurements, for nucleation of a droplet in a 90 nm NC subject to a field of 0.3 T. In order to confirm the correlation between the structure observed and the nano-contact position, a yellow dashed line denoting the edge of a domain formed in the vicinity of the nano-contact at −10 mA without external field (Fig. 5(a) is superimposed upon Fig. 5(b). Figure 5(c)and (d) are a close-up of the region enclosed within the red square in Fig. 5(b), where the black dashed line represents the possible position of a NC of 90 nm diameter.

We notice that the observed magnetic structure does not have the symmetrical circular shape expected of a droplet. It has been reported that canted magnetisation of the PL as well as the influence of the Oersted field^8,16,30^ might affect the stability of the droplet so that it may extend beyond the region immediately beneath the NC or even drift away from it completely. It is possible that Fig. 5(c) and (d) are showing the time averaged trajectory of the droplet as it deviates from the centre of the NC, but further measurements performed at different values of current and external field are needed to confirm this conjecture.

## Conclusions

The results shown in this paper are consistent with the local modification of the magnetisation around and beneath the NC due to the DC current. At zero field, and due to the size of the domains, we suggest that the modification of the magnetisation is due to the Oersted field produced by the DC current. When a small magnetic field is applied perpendicular to the sample plane, the interplay between the external field and the Oersted field modifies the size of the domains and erases them altogether when the external field becomes dominant, as can be seen in Fig. 4. Finally, when the external field and the DC current are set to 0.3 T and −35 mA respectively, the magnetic contrast shows a magnetic structure with reversed magnetisation which we suggest is a droplet soliton. Since the exact position of the NC is unknown it is not possible to state unequivocally that the observed magnetic structure lies beneath the NC. However, the size of the structure, its position relative to the domains induced at other DC current values, and the correlation with separate electrical measurements, suggest that the nucleation of a droplet soliton has occurred.

## Methods

### Sample fabrication

50 × 250 *μ*m^2^ rectangular patterns were exposed and developed on standard 4-inch thermally oxidized Si wafers using an optical lithography technique. A pseudo-spin-valve structure of composition Co (6 nm)/Cu (6 nm)/Co (0.2 nm)[Ni (0.8 nm)/Co (0.3 nm)] × 4, was then deposited by confocal magnetron sputtering onto a Ta (4 nm)/Cu (11 nm)/Ta (4 nm) seed layer and capped with a Cu (2 nm)/Pd (2 nm) bilayer. The lift-off process was completed by the removal of the remaining photo resist, and followed by the deposition of a 30 nm-thick SiO_2_ dielectric film by chemical vapour deposition. Nano-pores of different size were formed within the SiO_2_ using electron beam lithography and highly selective reactive ion etching. The device fabrication was completed by the deposition of a 400 nm Cu/100 nm Au top electrode using a combination of lift-off lithography and sputtering.

### Device Characterization

In order to electrically characterize the NC-STO, the sample was mounted in a custom probe station and a DC bias current from a Keithley 6221 current source was passed through the NC and between the signal and ground lines of the surrounding coplanar waveguide (CPW) structure. The current was swept from 0 to −40 mA under a fixed external field of 0.3 T at room temperature. In order to decouple and amplify the a.c. signal from the device, a bias-T and a 2–18 GHz AtlanTec(AS7265) amplifier were used respectively. The amplified a.c signal was recorded by an Agilent E448A spectrum analyser.

### HERALDO measurements

The measurements presented in this paper were taken on beamlines I06 and I10 of the Diamond Light Source using a continuous filling pattern (∼300 mA). To perform x-ray magnetic circular dichroism measurements on the samples, left and right-circularly polarised x-rays were applied with a photon energy of 851.1 eV coinciding with the Ni L_3_ absorption edge. The interference pattern of the direct and diffracted x-rays for each polarisation was recorded on a CCD camera positioned behind a beam stop, that blocked the direct non-diffracted beam, at a distance of 60 cm from the sample, resulting in a minimum spatial resolution of 32 nm. To obtain holographic images the sample had a reference slit that was isolated from the imaged area by a distance of twice the aperture diameter but remained within the x-ray transverse coherence length (∼25 *μ*m). Holograms recorded for left and right polarisations were subtracted and then processed using directional derivatives and Fourier transformed in order to obtain the real-space image.
